# Coalescence Dynamics of Acoustically Levitated Droplets

**DOI:** 10.3390/mi11040343

**Published:** 2020-03-26

**Authors:** Koji Hasegawa, Ayumu Watanabe, Akiko Kaneko, Yutaka Abe

**Affiliations:** 1 Department of Mechanical Engineering, Kogakuin University, Tokyo 163-8677, Japan; 2 Graduate School of Systems and Information Engineering, University of Tsukuba, Tsukuba 305-8573, Japan; awatanabelab@gmail.com; 3 Faculty of Engineering, Information and Systems, University of Tsukuba, Tsukuba 305-8573, Japan; kaneko@kz.tsukuba.ac.jp (A.K.); abe@kz.tsukuba.ac.jp (Y.A.)

**Keywords:** acoustic levitation, ultrasonic phased array, droplet, coalescence, lab-on-a-drop

## Abstract

The contactless coalescence of a droplet is of paramount importance for physical and industrial applications. This paper describes a coalescence method to be used mid-air via acoustic levitation using an ultrasonic phased array system. Acoustic levitation using ultrasonic phased arrays provides promising lab-on-a-drop applications, such as transportation, coalescence, mixing, separation, evaporation, and extraction in a continuous operation. The mechanism of droplet coalescence in mid-air may be better understood by experimentally and numerically exploring the droplet dynamics immediately before the coalescence. In this study, water droplets were experimentally levitated, transported, and coalesced by controlled acoustic fields. We observed that the edges of droplets deformed and attracted each other immediately before the coalescence. Through image processing, the radii of curvature of the droplets were quantified and the pressure difference between the inside and outside a droplet was simulated to obtain the pressure and velocity information on the droplet’s surface. The results revealed that the sound pressure acting on the droplet clearly decreased before the impact of the droplets. This pressure on the droplets was quantitatively analyzed from the experimental data. Our experimental and numerical results provide deeper physical insights into contactless droplet manipulation for futuristic lab-on-a-drop applications.

## 1. Introduction

The acoustic levitation [1,2] of droplets is a promising tool for contactless fluid manipulation for lab-on-a-drop applications [3,4,5,6,7,8] by achieving droplet levitation, transportation, coalescence, mixing, separation, evaporation, and extraction in a single operation. Through acoustic levitation, a sample can be suspended using the acoustic radiation force acting on the object [9]. This method enables contactless treatment to avoid the contamination, absorption, and heterogeneous nucleation through the wall effect [2]. In a study on single-acoustic levitation, Foresti et al. [10] examined the coalescence of droplets using multiple Langevin type transducers and reflectors. To develop acoustic levitation, Marzo et al. [11] demonstrated a novel technique for particle manipulation using an ultrasonic phased array system. Additionally, Andrade et al. [12] experimentally presented a manipulation method for a target sample for injection, levitation, transportation, coalescence, and ejection in mid-air. Watanabe et al. [13] investigated the mixing of droplets with an ultrasonic phased array system. After the successful merging of droplets, a single droplet was effectively mixed by inducing the 6th mode oscillation on its surface. Hasegawa et al. [14] observed that that the internal flow field induced by the mode oscillation promoted the mixing performance with varying Reynolds number.

Although contactless droplet manipulation in mid-air is vital for future lab-on-a-drop applications, the nonlinear effect of strong acoustic fields in acoustic levitation, such as interfacial instability [15,16] and its transport phenomena [17,18,19,20,21,22,23] have been partially understood to date. The dissemination of droplet manipulation in acoustic levitation using an ultrasonic phased array system requires the fundamental physics of a levitated droplet to be fully understood. While many studies have been conducted in the past decades to better understand acoustically levitated droplets, the levitation and coalescence stability of droplets remains unclear. 

In this paper, we present the experimental and numerical analyses of droplet coalescence in mid-air in acoustic levitation. To better understand the coalescence dynamics of droplets, we quantitatively investigated the characteristics of droplet deformation using image processing and the pressure on a droplet surface using numerical simulation. Our insights may lay the foundation for contactless droplet manipulation and stimulate further theoretical, numerical, and experimental studies.

## 2. Materials and Methods 

### 2.1. Experimental Design

**Figure 1:**
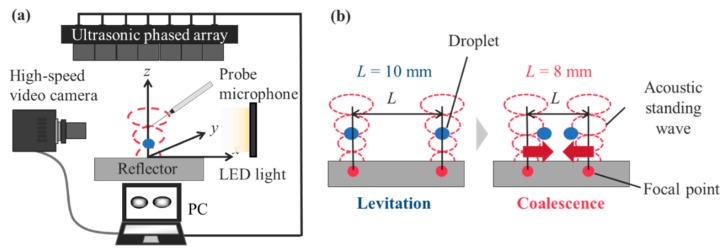
Schematic of the experimental setup and procedure: (**a**) Observation system with acoustic levitation. (**b**) Droplet coalescence procedure using an acoustic field. The droplets coalesced when the distance between them decreased from *L* = 10 to 8 mm.

A schematic of the experimental setup is presented in Figure 1a. The acoustic field for levitating and merging droplets was generated and controlled using an ultrasonic phased array [16]. A 40-kHz localized sound wave formed the focal point of the sound. The acoustic potential enabled us to trap droplets near the pressure node of an acoustic standing wave. Forty nine small ultrasonic transducers (7 × 7 square arrangement) controlled the phase of the sound wave using a field-programmable gate array (Cyclone-IV DE0-Nano, Intel Corp., Santa Clara, CA, USA). Each transducer was 10 mm in diameter. The transducer-reflector distance was 45 mm. The rapid switching of two focal points at a frequency of 500 Hz enabled two droplets to levitate simultaneously. After the successful levitation of two droplets, the distance between two focal points was decreased from 10 to 8 mm to coalesce the droplets. For decreasing the distance between two focal points, the time delay of the propagating wave front of the generated sound was controlled by tuning the phase of the signal from each transducer. The initial droplet diameter before a coalescence ranged from 1.0 to 2.0 mm. The root-mean-square (RMS) sound pressure was maintained at approximately 1.7 kPa in an ambient temperature of 20 °C. The sound pressure measurement was conducted using a probe microphone (Type 4138, diameter: 3.175 mm, Brüel & Kjaer, Narum, Denmark). The motion of the droplets was captured via a high-speed video camera (FASTCAM-Mini UX100, Photron Co., Ltd., Japan) using a backlight method (OPF-S77×77W-PS, Optex FA Co., Ltd., Tokyo, Japan). The test fluid was pure water. The uncertainty of the droplet diameter was less than 3% because minimum levitated droplets of 1.0 mm were observed with a spatial resolution of ≈15 μm/pixel, and the standard deviation with three measurements was less than 2 pixels.

### 2.2. Numerical Simulation

To better understand the experimental data, we simulated the sound field of the ultrasonic phased array system using the distributed point source method (DPSM) [16,24]. DPSM was used to describe the acoustic field with computational meshfree methods by distributing the point sources of sound at the boundary. Ultrasonic transducers and reflectors were discretized by point sources to simulate the sound field. The sound pressure, *p_m_*, and velocity, *v_m_*, at a distance, *r_mn_*, generated by the sound wave from the point source m can be expressed as follows:

(1) $$p_{m}\left(r_{\mathrm{mn}}\right)=A_{m}\frac{\exp i\left(kr_{\mathrm{mn}}-\omega t_{m}\right)}{r_{\mathrm{mn}}},$$

(2) $$v_{m}\left(r_{\mathrm{mn}}\right)=\frac{n\cdot r_{\mathrm{mn}}}{i\omega \rho_{0}}\frac{\partial p}{\partial r}=A_{m}M\left(r_{\mathrm{mn}}\right),$$

where *A_m_* is the sound amplitude of the *m*th point and *ω* is the angular frequency. For the ultrasonic phased array, sound waves emitted from each transducer were overlapped after different time intervals Δ*t_m_* to focus the sound waves. Subsequently, the term *t_m_* in Equation (1) can be described by a phase difference from a reference time *t* which is determined by the simulation geometry.

For *N* point sound sources, the total sound pressure and velocity at point *x* can be derived as follows:

(3) $$p\left(x\right)=\overset{N}{\underset{m=1}{\sum }}p_{m}\left(r_{\mathrm{mn}}\right),$$

(4) $$v\left(x\right)=\overset{N}{\underset{m=1}{\sum }}v_{m}\left(r_{\mathrm{mn}}\right).$$

When secondary sound sources such as reflectors exist, the amplitudes of the sound are determined using the boundary conditions. For an unknown amplitude, *N* point sources are distributed for both primary (e.g., transducers) and secondary sound sources (e.g., reflector). Here, the sound sources were assumed to be spheres of radius *r_s_*. In addition, the radiation points were assumed to be displaced by *r_s_* from the boundary. With a non-zero velocity *V_0_* for the primary sound sources and a zero velocity for the secondary sources, the following equations can be derived:

(5) $$\left\{v_{m}\right\}={}^{t}\{V_{0},\;\cdots V_{0},\;0,\;\cdots 0\},$$

(6) $$\left\{A_{m}\right\}={\left[M_{\mathrm{mn}}\right]}^{-1}\left\{v_{m}\right\}.$$

Here, *N* equations were obtained for *N* unknown sound sources. From the abovementioned numerical framework, the velocity and amplitudes of the unknown sound sources were derived.

## 3. Results and Discussion

### 3.1. Experimental Analysis: Visualization of Droplet Coalescence in Mid-Air

**Figure 2:**
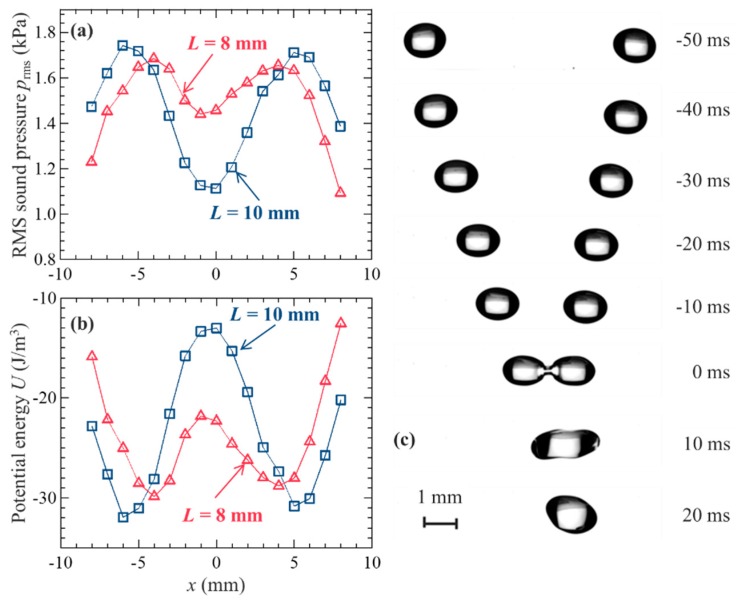
Coalescence procedure and visualization of droplets: (**a**) RMS sound pressure and (**b**) acoustic potential energy distributions for *L* = 8 and 10 mm, as shown in Figure 1b. (**c**) Droplet coalescence procedure in an acoustic field. The droplets coalesced when the distance between them decreased from *L* = 10 to 8 mm; 0 ms indicates the moment the droplets make contact.

Prior to the coalescence of the droplets, the sound pressure distribution on the pressure nodal plane between the transducer-reflector distances was measured. Figure 2a shows the RMS sound pressure distribution *p_rms_* for different distances, *L*, between two focal points. For *L* = 10 mm (initial condition), a clear distribution with higher and lower sound pressure was observed. Two droplets could be trapped by the generated sound pressure difference. *L* was decreased to 8 mm to coalesce the droplets. Hence, the sound pressure distribution was distorted and the sound pressure at approximately *x* = −1 mm increased. The characteristics of the sound wave were better understood by quantifying the acoustic potential energy as follows [25]:

(7) $$U≅\frac{p_{rms}^{2}}{\rho_{a}c^{2}}\left\{-\frac{3}{2}+\frac{5}{2}\cos^{2}\left(\frac{2\pi z}{\lambda }\right)\right\},$$

where *p_rms_* is the RMS pressure of the surrounding fluid near the pressure node, *ρ_a_* is the density of air, *c* is the speed of sound, *z* is levitation height of 3/4*λ*, and *λ* is the wavelength of a sound wave of 8.5 mm. Figure 2b represents the acoustic potential energy distribution from Figure 2a. For *L* = 10 mm, the figure indicates that droplets could be trapped around the lower acoustic potential energy at approximately *x* = ± 5 mm. When the distance between the focal points decreased to 8 mm, the acoustic radiation pressure acting on the droplets generated an inertial force and pushed droplets to the center, and they coalesced. 

Figure 2c shows the typical visualization of droplets coalescing in the acoustic field. A pair of millimeter-sized water droplets were simultaneously and stably levitated at –50 ms. The droplets were forced to move toward the center by the movement of the focal point of sound from *L* = 10 to 8 mm, as mentioned above. At 0 ms, the droplets began to merge and successfully coalesced. However, our previous study revealed that droplets cannot coalesce under certain conditions. For larger sound pressure and droplets, the droplets can disintegrate or fall immediately after coalescence [16]. Droplet atomization can result from the interfacial instability [26] on a droplet surface with higher sound pressure. The droplets fell because larger droplets could not counteract the weight of the coalesced droplet. These results indicated that precise tuning of both droplet size and sound pressure was required, and the physical conditions for successful coalescence needed to be explored.

**Figure 3:**
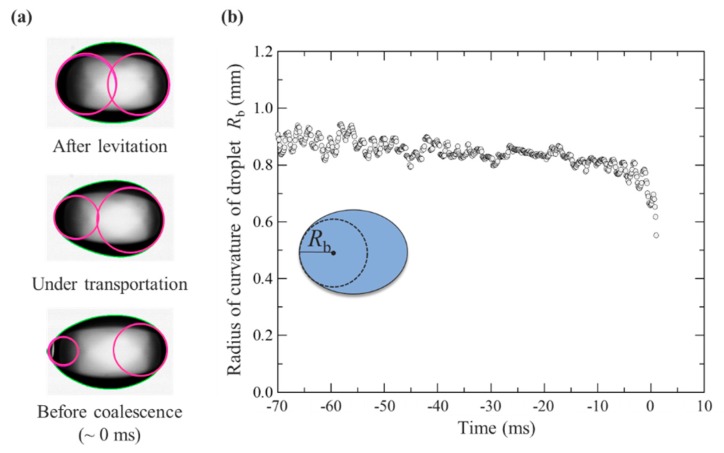
Quantification of droplet motion before the coalescence shown in Figure 2c: (**a**) Image processing of droplet interface. (**b**) Time evolution of the radii of curvature of droplets (left edge) from levitation to coalescence. The inset represents the schematic description of the radius of curvature of a droplet.

For a better understanding of the coalescence dynamics on the levitated droplet, we quantified the time evolution of the radius of curvature of the droplet. Figure 3 shows the quantification of the droplet motion before coalescence based on Figure 2c. Original images were processed using in-house codes with the following procedure: (1) interface detection, (2) fitting of the droplet interface using the least square method, and (3) the best-fitted circle was used as the best approximation and the radius of curvature of the droplet. Figure 3a represents the typical images of a droplet after levitation (top), under transportation (middle), and immediately before coalescence (bottom). The pink circles in the droplet indicate the radii of curvature of droplets using image processing. The droplet shapes deformed during the transportation and their radii of curvature were minimized before the droplets coalesced. Figure 3b represents the time evolution of the radii of curvature of the droplets_._ These results depict the left edge of a droplet interface. The radii of curvature slightly decreased during transportation from –70 to approximately –5 ms. Immediately before the contact of the droplets, their radii of curvature abruptly decreased from 0.8 to 0.6 mm. This nonlinear behavior contributed significantly to droplet coalescence. This was considered due to the interaction of droplets and sound pressure field when the droplets were closely approaching.

### 3.2. Numerical Simulation: Comparison with the Experiment

**Figure 4:**
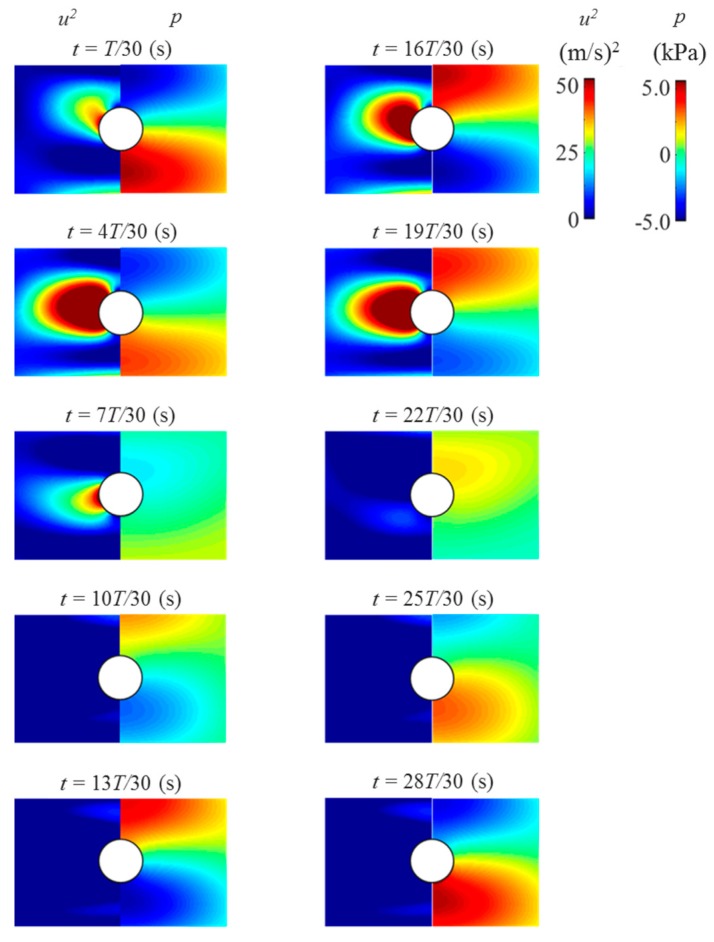
Time evolution of velocity (**left**) and pressure (**right**) fields around the levitated solid sphere using DPSM. The solid sphere was displaced 1 mm below the pressure node due to the gravitational effect to simulate the experimental result.

In the experiment, the acoustic radiation pressure exerted on the acoustically levitated droplets could not be measured. A numerical simulation using DPSM was conducted to identify the velocity and sound pressure fields around the droplet. Before the simulation of the approaching droplets, Figure 4 depicts the instantaneous velocity and pressure fields around the levitated solid sphere for the validation of the numerical simulation. The radius of the solid sphere was 1 mm. In the figure, *t* is instantaneous time and *T* is the duration of time of one cycle. From the velocity fields around the solid sphere, the velocity fluctuation in the vicinity of the solid sphere was observed to be larger than its surroundings and the effect of the velocity field could be more dominant near the droplet interface. For the sound pressure fields around the solid sphere, the sound pressure fluctuated above and below the solid sphere, which expressed the antinodes and nodes of the sound pressure. The levitated droplet near the pressure node was suppressed from above and below by the sound pressure field and pulled from the side. Based on these simulations, the acoustic radiation pressure *p_rad_* on the droplets can be expressed as

(8) $$p_{\mathrm{rad}}=\frac{p_{rms}^{2}}{2\rho_{a}c^{2}}-\frac{\rho_{a}u_{rms}^{2}}{2},$$

where *u_rms_* is the RMS velocity of surrounding fluid near the pressure node. This simulation exhibited positive agreement with the experimental results. Thus, these results indicate that DPSM can be used to simulate the physical phenomena of acoustically levitated droplets.

**Figure 5:**
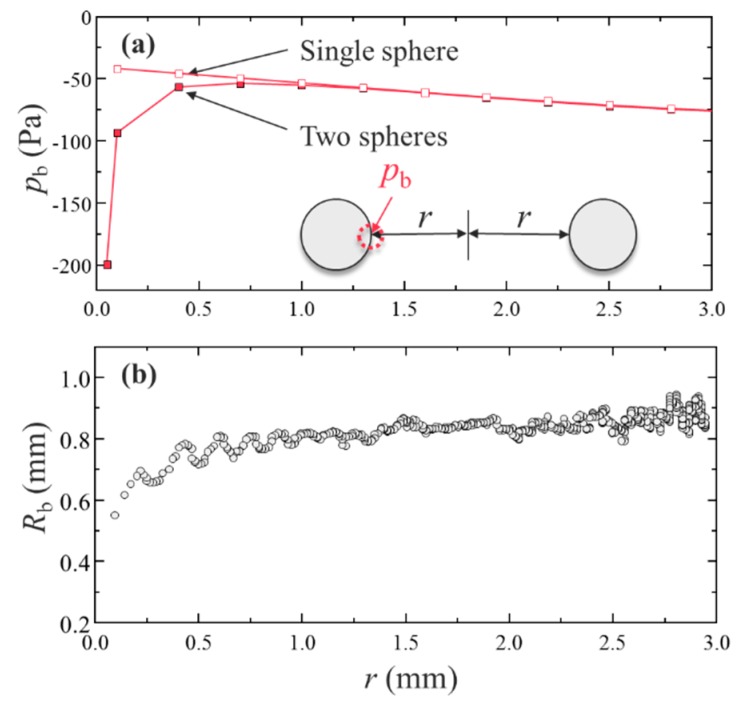
Acoustic radiation pressure at the edge when approaching a solid sphere: (**a**) Acoustic radiation pressure on the droplet edge using DPSM for a single and two solid spheres. The single sphere result is overlapped for comparison. (**b**) Droplet radii of curvature (left edge) from levitation to coalescence as a function of the distance between droplets obtained from Figure 3b.

**Figure 6:**
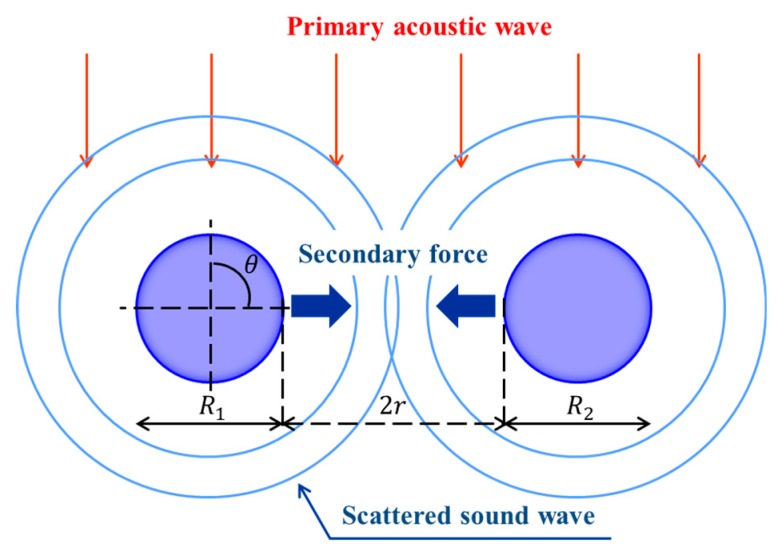
A schematic illustration of the secondary force in the present study. The secondary force on the droplets was calculated by assuming *θ* = 90°.

Figure 5a presents the acoustic radiation pressure at the edge of droplet *p_b_* during the transportation as a function of the distance between droplets *r*. Here, *r* is the distance from the droplet collision point. To compare the experimental analysis with the simulation, Figure 5b describes the radius of curvature of a droplet (left edge) from levitation to coalescence. Figure 5a indicates that when the solid spheres were approaching, the acoustic radiation pressure significantly decreased before the collision (*r* < 1.0 mm). Figure 5b confirms the same tendency of the radius of curvature of the droplet (*r* < 1.0 mm). This simulation verified the experimental analysis in Figure 5b. A reason for this could be the secondary force triggered by the interaction between the approaching droplets. Although droplets were levitated and transported by the primary acoustic radiation force, when the droplets became significantly closer (within submillimeter distance), the secondary force generated by the scattering of the primary acoustic field [10,27] became dominant. Assuming that the density of the spheres is significantly greater than that of air, *ρ*_a_, the secondary force on the droplets, *F_r_*, if the angle between the directions of wave propagation and the axis connecting two spheres is *θ* = 90°, can be calculated as

(9) $$F_{r}≅\frac{-3\pi \rho_{a}R_{1}^{3}R_{2}^{3}u_{rms}^{2}}{{\left(2r\right)}^{4}}\;$$

where *R_1_* and *R_2_* are the radii of the two spheres. A schematic of the secondary force is illustrated in Figure 6. For *R_1_*/*R_2_* of 1 mm, *u_rms_* of ≈0.3 m/s, and *r* of 0.1 mm for both spheres, the secondary force on the droplets was approximately −0.6 mN. The acoustic radiation pressure on the droplet can be –190 Pa when the surface area of droplet *A* (=π*R*^2^) is ≈3.14 μm^2^. This estimation indicates an excellent agreement with our simulation result of −200 Pa at approximately 0.0 ms, as shown in Figure 5a. Additionally, the surface tension of the droplet can play an important role in the dynamics of the coalescence, as shown in Figure 3a. From Figure 5b, the droplet deformation by attractive secondary force can be expressed by the Laplace pressure. For the surface tension of water of ≈70 mN/m and the minimum radius of curvature of a droplet *R_b,min_* of ≈0.5 mm, the Laplace pressure on the droplet, ≈ σ/*R_b,min_*, is roughly ≈140 Pa (by calculation). This estimation by the Laplace pressure shows the same order of the pressure difference obtained by the numerical simulation. Another possible reason for the sensitive coalescence dynamics could be the complex interfacial instability of the droplets; however, the exact reason remains a challenge because when the droplets collide, unsteady interfacial behaviors can be possibly triggered and developed, which are difficult to directly measure. These sensitive coalescence dynamics may strongly affect the droplet instability by driving the fluctuation of unsteady flow and sound pressure fields between the approaching droplets. Although the numerical simulation in the present study treated the droplet as a solid sphere without shape changes, the instantaneous interfacial motions of droplets, the pressure distribution among attracting droplets, and the effect of the flow field in a droplet need to be explored by improving the numerical simulation in our future research. Furthermore, the diffusion and reaction phenomena before and after the successful coalescence of droplets are also promising and will be experimentally and numerically investigated for a futuristic droplet manipulation. 

## 4. Conclusions

In this study, we experimentally and numerically demonstrated the coalescence dynamics of acoustically levitated droplets using an ultrasonic phased array system. The coalescence dynamics of droplets in mid-air were fully understood by experimentally and numerically exploring the water droplets immediately before the coalescence. We observed that the edges of the droplets deformed and attracted each other immediately before the coalescence by quantifying the radii of curvature of droplets via in-house image processing. The symmetrical droplet shape contributed significantly to the coalescence of droplets. This may be have due to the interaction of droplets and sound pressure field when the droplets were closely approaching with less than a millimeter of the distance between them. To identify the physical mechanism of the droplet coalescence, we simulated the pressure and velocity fields around the levitated solid sphere using DPSM. The time evolution of the sound pressure between droplets was obtained to elucidate the experimental data. The simulation result indicated that the acoustic radiation pressure exerting on the droplet drastically decreased before the droplets collided. This pressure change on the droplets immediately before the coalescence was approximately analyzed using the secondary force on the droplets by the scattering of the primary acoustic field and this indicated positive agreement with our experimental demonstration. Our experimental and numerical results present clear physical insights into the dynamics of contactless droplet coalescence. Further investigation is required to explore the optimum conditions of droplet coalescence, including the best speed of collision and the combination of liquid properties of the droplets. A clear understanding of droplet dynamics during contactless droplet manipulation via acoustic levitation will provide better physical and application insights for potential lab-on-a-drop applications [28,29,30].
